# Food and nutrition information requirements of Australian primary school parents

**DOI:** 10.1017/S1368980024000387

**Published:** 2024-02-05

**Authors:** Gozde Aydin, Claire Margerison, Anthony Worsley, Alison Booth

**Affiliations:** 1 Institute for Physical Activity and Nutrition (IPAN), School of Exercise and Nutrition Sciences, Deakin University, Geelong, Australia; 2 School of Exercise and Nutrition Sciences, Deakin University, Melbourne, Australia

**Keywords:** Parents, Primary school, Food and nutrition, Food literacy

## Abstract

**Objective:**

To explore what Australian primary school parents want to learn about food and nutrition to improve their children’s eating behaviours, as well as the associations between parents’ personal and demographic characteristics and their views regarding their food and nutrition knowledge needs.

**Design:**

An online nationwide cross-sectional survey was conducted in 2021 using a mixed-methods approach. Logistic regression analysis was utilised to examine the relationship between parents’ demographics, personal values and their views. Content analysis was performed using Leximancer.

**Setting:**

Australia.

**Participants:**

Seven hundred and eighty-seven parents.

**Results:**

Fifty-one per cent wanted to learn more about food and nutrition to improve their children's healthy eating habits, and 77% of those preferred schools to provide that information. Online/printed newsletters and YouTube were the most preferred methods for receiving food and nutrition related information. Higher universalism-concern value (concern for the welfare of those in the larger society and world) scores were positively associated parents’ preference for schools to provide food and nutrition-related information. Parents with non-English-speaking backgrounds and younger parents were more likely to want to learn about food and nutrition. Parents wanted to learn more about encouraging healthy eating, ideas for the lunchbox, food labels and age-specific portion sizes and recommendations.

**Conclusions:**

Findings can inform public health educators and assist them in designing future food and nutrition education programmes and resources targeting primary school parents.

Globally, many primary school-aged children do not consume the diets they require, hindering their capacity to grow, develop and achieve their full learning potential^(1)^. In Australia, a significant number of children do not consume adequate amounts of fruits and vegetables, and approximately 40 % of their daily energetic intake is comprised of nutrient-poor, energy-dense foods^(2)^. These unhealthy eating habits can carry into adulthood^(3)^, increasing the risk of non-communicable chronic diseases such as CVD and type 2 diabetes^(4)^. A suboptimal diet can also result in psychological and social issues, such as the stigma associated with obesity,^(5)^ and potentially decrease children’s academic performance^(6)^.

The family environment is considered the primary factor that shapes young children’s diets, as parents are responsible for supplying and preparing the majority of their food during the early years. Parental behaviour serves as a model for their children’s food choices within the home, and demonstrating healthy eating habits can greatly impact children’s consumption of nutritious foods^(7)^. In addition, previous studies have found a strong positive relationship between parental nutrition literacy and children’s diet quality^(8)^, and children’s nutrition knowledge^(9)^. As parents play a crucial role in the formation children’s eating habits and food literacy, examination of their experiences in feeding their children is imperative.

Any programme aiming to improve young children’s diets needs to assist parents in overcoming their barriers to ensuring healthy eating for their children. To be able to design such programmes, it is imperative to do a needs assessment and explore parents’ views regarding the programme content and delivery methods. However, to date, only a few studies have investigated primary school parents’ food and nutrition information-related needs^(10–12)^. For example, in Slusser’s study, primary school parents from low-income backgrounds in the USA expressed their desire to acquire knowledge on how to select healthy foods, prepare nutritious meals, encourage their children to eat healthily and interpret food labels^(10)^. In Hart’s study, British parents, particularly those from lower socio-economic backgrounds, expressed a need for more information about the sources of nutrients and practical cooking skills^(11)^. To fill these research gaps, especially in the Australian context, this study explored what Australian primary school parents want to learn about food and nutrition to improve their children’s eating behaviours and the ways they prefer to learn.

The present study also explored the associations between parents’ personal and demographic characteristics and their views regarding their food and nutrition information needs. Previous studies have shown that demographic characteristics such as educational background and age can impact food-related views and expectations^(13)^. It also is extensively documented that personal values play a pivotal role in consumer behaviour, particularly in domains like food behaviour, as illustrated in the Food-related Lifestyle Model^(14)^. Specifically, universalism and hedonism values have been associated with food and nutrition-related views and expectations^(15)^. Thus, we hypothesised that the food and nutrition knowledge needs of parents would be related to their demographic characteristics and personal values scores.

## Methods

### Design and sampling

The study adopted a mixed-method approach. The quantitative part of the study aimed to identify consistent patterns and demographic associations of parents’ views, whereas the qualitative component revealed complex concepts that may not be captured by closed-answer questions^(16)^. We adopted a critical realist approach, acknowledging that although there is a reality to observe and document, the research still reflects the perspectives of the researchers^(17)^. Within this approach, we employed a descriptive, theoretical framework to explore parents’ views. Descriptive qualitative studies provide an overview of a phenomenon in everyday words as stated by the participant rather than interpreting their responses^(18)^. We adopted this approach as it was the most feasible way of analysing the ‘thin’ data collected, where parents provided brief open-ended responses to the questions.

A cross-sectional survey conducted online using the Qualtrics platform was employed to obtain parents’ views of primary school food environments, practices and policies. To be eligible to participate in the survey, respondents had to reside in Australia and be parent/primary carers of a child attending an Australian primary school at the time of the study. Both paid and unpaid recruitment strategies were utilised on social media platforms such as Facebook and Twitter to attract respondents. To incentivise participation, respondents were given the opportunity to win one of five $50 gift vouchers upon completion of the survey.

#### Survey questionnaire

The survey consisted of thirty-one closed-ended questions with five sub-questions and seven open-ended questions. The authors’ previous qualitative research on the views of parents and teachers regarding food and nutrition education and environments in primary schools^(19,20)^ and relevant literature on school food environments informed the development of the questionnaire. The present paper reports the results from three closed-ended and one open-ended question that focused specifically on parents’ food and nutrition information needs (Table 1). Parents were asked whether they would like to learn more about food and nutrition to improve their child/children’s healthy eating habits. If they responded ‘Yes’, they were asked what specifically they would be interested to learn more about. Then, these respondents were also asked how they would like to receive food and nutrition-related information and whether they would like their child’s primary school to provide information for them. Additional details about the survey design have been reported previously^(21,22)^.

**Table 1:** Survey questions and response options

Questions	Response options
1. Would you like to learn more about food and nutrition to improve your child/children’s healthy eating habits?	Yes/no/unsure
What specifically would you be interested to know more about?	Open-ended
How would you like to receive food and nutrition-related information? Tick all that apply.	Several options were listed: • In-person workshops during school hours; • In-person workshops after school hours; • Printed newsletters/brochures/books; • Webinars that are interactive with a facilitator; • Online newsletters/brochures/book; • YouTube clips that discuss a food, nutrition or health science topic; • Podcasts that present information by food, nutrition and health scientists and educators; • Online discussion forums conducted at a common time with a facilitator.
Would you like your child’s primary school to provide information for you?	Yes/no

Note: Websites/social media were not explicitly listed as a response option.

#### Personal values

This paper also included data from the personal values section of the whole survey to investigate the associations between parents’ views and their personal values. Nine items were chosen from the fifty-seven-item Schwartz Personal Values inventory^(23)^, including values of universalism-nature, universalism-concern and hedonism (three items per value). Universalism nature is a value related to motivation for the preservation of the natural environment and animals, whereas people who hold universalism-concern values prioritise principles such as equality, justice, compassion and the common good for all members of society^(23)^. These values have been shown to be associated with people’s food-related practices and beliefs^(13)^. Previous research has demonstrated that individuals with high universalism values tend to consume healthier food^(13)^ and support healthy eating policies^(24)^ and initiatives that promote fruit and vegetable consumption^(25)^. In line with these previous findings, we expected parents with high universalism values to be more interested in receiving food and nutrition information, whereas hedonists were expected to be less interested.

Parents were asked: *‘To what extent do the following statements describe you and your approach to life*?’ and were asked to rate the importance of each item on a five-point Likert scale. The internal reliability for each personal value was measured and found to be 0·79 for hedonism, 0·80 for universalism-concern and 0·82 for universalism-nature. The respondents’ personal value scores were calculated by taking the mean of the ratings given to the three items for each personal value.

#### Demographic characteristics

The survey also included six questions on demographic information such as gender, age, marital status, highest level of education attained, main language spoken at home and residential postal code. The geolocation was determined based on the residential postcode utilising the Accessibility and Remoteness Index of Australia (ARIA+)^(26)^. Socio-economic status (SES) was identified by mapping the residential postcodes to the Socio-Economic Indexes for Areas (SEIFA)^(27)^. In this study, population groups in deciles 1–3 were defined as ‘low SES’, while population groups in deciles 8–10 were defined as ‘high SES’.

#### Survey administration

Before taking the survey, parents were provided with a Plain Language Statement available for download. They were then asked if they had read the statement and consented to participate in the study. A pre-test of the entire survey was carried out to identify any issues with the wording and structure of the questions. Nine parents who were not part of the final study participated in the pre-test^(28)^. Following the pre-test, two sections were removed to shorten the completion time to approximately 15–20 min, and minor modifications were made to the wording and sentence structure of the questionnaire. The survey was conducted between March and April 2021. The Deakin University, Faculty of Health, Human Ethics Advisory Group granted ethics permission for this study.

## Data analysis

The data obtained from the closed-ended questions in the survey were analysed using IBM SPSS version 27 software. Descriptive statistics were calculated, and forward logistic regression analyses were performed to identify variables that may be associated with parents’ views. The variables tested were gender (female *v*. male), parental age (continuous variable), main language spoken at home (English *v*. other), parental education level (postgraduate degree, university degree, Year 12 or less) SES (high, mid and low), geolocation (living in rural *v*. major city), child’s grade and parents’ universalism-concern, universalism-nature and hedonism value scores. Before conducting logistic regression, to assess multicollinearity, Tolerance values (ranging from 0·74 to 0·94) were examined, and as they exceeded 0·1, and Variance Inflation Factor (VIF) values (ranging from 1·06 to 1·34) were below 10, no correlations were identified between variables^(29)^. A two-sided type 1 error of 0·01 was considered a significant difference.

**Fig. 1:**
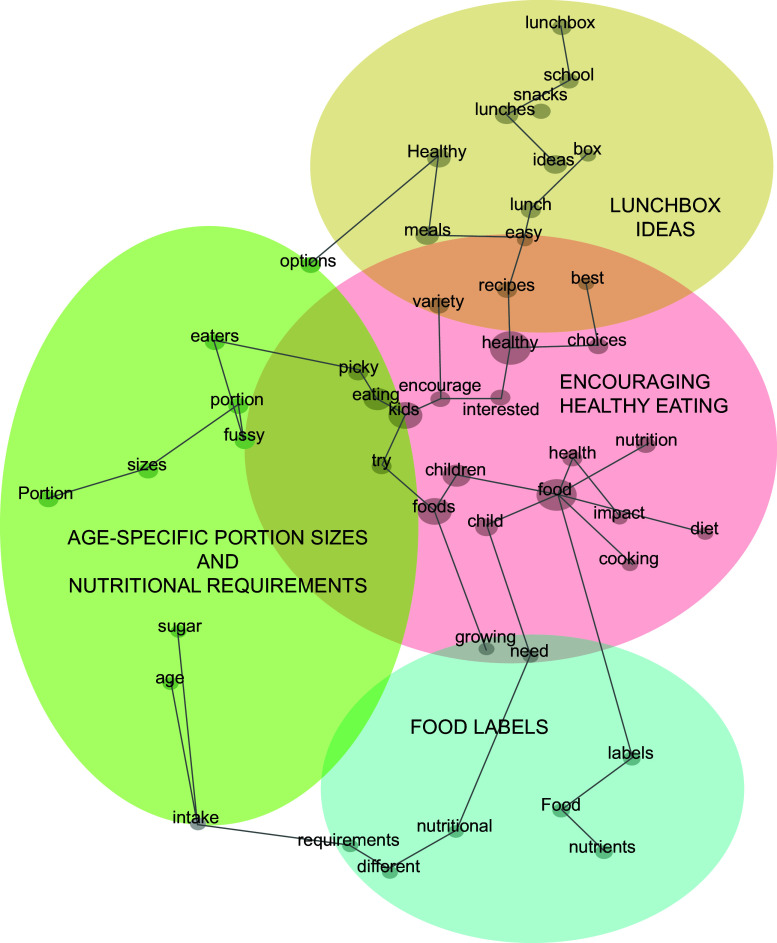
Leximancer map of parents’ food and nutrition information requirements

The responses to the question ‘What specifically would you be interested to know more about?’ were loaded into the Leximancer software (version 5, Leximancer Pty Ltd, 2021). This machine learning-based qualitative data analysis tool analyses text data to automatically identify and categorise concepts and themes based on word frequencies and co-occurrence^(30,31)^. In the final analysis step, these identified themes and concepts were visualised in a concept map (Fig. 1). The large circles represent the identified themes, whereas the dots represent the concepts. Leximancer labels the most prominent concepts as themes based on their interconnections with other concepts^(32)^. The themes were also ‘heat mapped’ to demonstrate their relative connectivity with the other concepts. The hot colours, like red and orange, symbolised the most important themes, while cool colours, such as blue and green, represented less significant themes^(33)^. The themes generated by Leximancer were manually renamed to provide more meaningful names through a process of multiple readings of the comments associated with each theme. However, it is important to note that the analysis was not solely based on the software’s output, as the researcher should also perform manual sensemaking to ensure that the findings are meaningful and insightful^(34)^. During analysis, the first author read the quotes from the respondents under each theme and worked with co-authors to develop narratives of the themes identified.

## Results

### Demographic characteristics of the respondents

Out of the 1259 individuals who opened the survey link, 787 completed it, resulting in a completion rate of 62 %. The parents represented a wide range of age, education, geolocation and socio-economic level categories. Nearly, all of the respondents were female (96 %), married (86 %) and had at least a university degree (72 %). The mean age of the respondents was 40 years. Although the survey was conducted across Australia, over half of the respondents were from the state of Victoria (56 %). In total, 66 % of the parents were from major cities, similar to the distribution in the Australian population, where 71 % lived in major cities^(35)^. The primary language spoken at home was English (93 %), and the majority of respondents were from high SES backgrounds (54 %) or mid-SES backgrounds (37 %). The sample was similar to the Australian population in terms of the proportion living in major cities and the proportion born in Australia. However, the sample was skewed towards high and mid-SES and more university-educated compared with the Australian average^(36)^. A table outlining demographic characteristics is provided in the supplementary file, and detailed information can also be found in a previous publication^(21)^.

### Parents’ responses to closed-ended questions

Approximately half of the parents (51 %) wanted to learn more about food and nutrition to improve their child/children’s healthy eating habits. These parents were then asked the following questions, ‘What specifically would you be interested to know more about?’, ‘How would you like to receive food and nutrition-related information? and ‘Would you like your child’s primary school to provide information for you?’. Seventy-seven per cent of those wanted their child’s school to provide that information. Online and printed newsletters and YouTube were the most popular methods to receive food and nutrition-related information, whereas workshops on weekends was the least preferred (Table 2).

**Table 2:** Parents’ food and nutrition information needs

Would you like to learn more about food and nutrition to improve your child/children’s healthy eating habits? (*n* 787)		
	*n*	%
Yes	395	51 %
No	269	34 %
Unsure	123	17 %
How would you like to receive food and nutrition-related information? Tick all that apply (*n* 394)		

	*n*	%
Online newsletter	225	57 %
Printed newsletter	209	53 %
YouTube	204	52 %
Webinars	156	40 %
Podcast	150	38 %
Workshop after school	111	28 %
Discussion forum	96	24 %
Workshop on weekends	51	13 %
Would you like your child’s primary school to provide information for you? (*n* 392)		

	*n*	%
Yes	303	77 %
No	89	23 %

### Variables associated with parents’ wanting to learn about food and nutrition to improve their children’s healthy eating habits

The stepwise logistic regression model included two statistically significant independent variables. First, non-native English-speaking parents were three times as likely as native-English-speaking parents to want to learn more about food and nutrition (*P* < 0·001). Second, for every 1-year increase in parents’ age, the odds of wanting to learn more about food and nutrition decreased by 3 % (*P* = 0·008) (Table 3). Although it was not significant for the set *P*-value, for every unit increase in the universalism-nature value score the odds of wanting to learn more about food and nutrition increased by 24 % (*P* = 0·03).

**Table 3:** Associated factors of parents wanting to learn more about food and nutrition to improve their child/children’s healthy eating habits

(*n* 392)	Parents’ wanting to learn more about food and nutrition to improve their children’s healthy eating habit			Parents’ wanting to receive food and nutrition-related information from their child’s school		
Variable	OR	95 % Cl	*P*	OR	95 % Cl	*P*
Universalism-concern score				1·99	1·37–2·91	<0·001
Parental age	0·97	0·943–0·991	0·008			
Main language spoken at home (Other *v*. English)	2·99	1·63–5·49	<0·001			
Universalism-nature score	1·24	1·02–1·50	0·034			

### Variables associated with parents’ wanting to receive food and nutrition-related information from their child’s school

The stepwise logistic regression model included only one independent variable, which was the parents’ universalism-concern value score. Higher universalism-concern value scores were associated with higher odds of parents’ wanting to receive food and nutrition-related information from their child’s school. For every unit increase in the universalism-concern score, the odds of parents wanting to receive food and nutrition-related information from their child’s school increased by 99 % (Table 3).

### Parents’ responses to the open-ended question: what do parents want to learn to improve their children’s eating behaviours?

The Leximancer analysis helped identify four themes which are explained in detail below. Parents wanted to learn more about encouraging healthy eating, ideas for the lunchbox, food labels and age-specific portion sizes and recommendations. The identified concepts and themes are presented in Fig. 1.

#### Theme 1: encouraging healthy eating (Leximancer label: healthy)

Parents’ responses indicated that many were interested in finding effective new ways to encourage their children to eat healthily and try new types of food.

*‘How to keep my kids interested in healthy eating as they get older and how to encourage my child to eat healthily at lunchtimes/recess.’ Parent 123*

Parents reported that they occasionally found it difficult to influence their kids to make healthier dietary choices. They frequently used the words ‘picky’ and ‘fussy’ as seen on the concept map (Fig. 1).

*‘When my child is picky with the food they eat, how to improve their diet in a mentally healthy and long-lasting way.’ Parent 329*

Parents wanted to know the appropriate language to use while communicating with their children about healthy eating due to their concerns about damaging their children’s relationship with the food.

*‘How to safely highlight eating habits without emphasis on weight/obesity and eating disorders’ Parent 678*

Finally, parents were keen to learn more about easy and healthy recipes to attract their children to be more involved in cooking.

*‘Easy recipes that are healthy that my children can easily prepare themselves’ Parent 299*

#### Theme 2: ideas for the lunchbox (Leximancer label: ideas)

This theme was the second most prominent and indicated parents’ willingness to receive lunchbox ideas to improve their children’s healthy eating. Parents wanted easy, healthy and budget-friendly lunchbox recipes and ideas for both snacks and lunches, as seen on the concept map (Fig. 1).

*‘Quick and easy lunch box recipes that the kids will eat that won’t break the budget’ Parent 702*

They also referred to the barriers they face while preparing their children’s lunchboxes, such as the high prevalence of allergies among school-aged children in Australia, which limits lunchbox options and the limited time given to children to eat their lunch.

*‘How to provide a balanced lunchbox when unable to include food groups due to school’s banning child’s preferred fruits, vegetables and protein(eggs) due to others’ allergies.’ Parent 453*

*‘How to diversify meals as much as possible, especially for school lunch boxes that need to be eaten in less than 15 min.’ Parent 227*

They were not only interested in the content of the lunch box, but in addition, some were curious to know how to preserve the food in the lunch box and its nutritional value.

*‘How to pack lunch box properly so that food does not lose its nutritious value’ Parent 340*

#### Theme 3: food Labels (Leximancer theme: labels)

This theme revealed that parents were keen to know more about food labels and how to read them to improve their children’s healthy eating. Some parents believed that by being able to read food labels better, they would make healthier choices while buying pre-packaged food. The word ‘label’ was frequently mentioned together with the word ‘requirement’, which indicates that parents who were interested to know more about labels were also interested in meeting nutritional requirements.

*‘Making healthy choices when purchasing pre-packaged foods/reading food labels.’ Parent 569*

*‘How to read labels and the actual nutritional value of different foods’ Parent 191*

#### Theme 4: age-specific portion size and nutritional requirements (Leximancer labels: size and age)

Theme 4 was created by merging two Leximancer themes, Size and Age. Parents demonstrated their interest in knowing more about the recommended portion of food for their child’s age and activity level.

*‘Appropriate portion sizes for young children, I never know if my kids are eating enough’ Parent 604*

Some stated their curiosity about nutritional requirements for certain age levels, specifically for their children. For example:

*‘A visual schedule of what a child would need to eat in a day to meet their daily requirements for key nutrients such as iron and vitamin D, calcium’ Parent 500*

## Discusssion

The current study explored parents’ food and nutrition-related knowledge requirements, as well as the variables of their views. To the best of the authors’ knowledge, this is the first study that has examined Australian primary school parents’ views on this topic. About 50 % of the surveyed parents expressed a desire to learn more about food and nutrition to improve their children’s healthy eating habits. These parents, when asked if they would like their child’s primary school to provide such information, over 75 %, responded positively. It is essential to acknowledge that those parents who were uncertain or resistant to furthering their knowledge about food and nutrition were not asked about their preference for receiving information through schools. Consequently, the overall percentage of parents eager to obtain resources from schools might have been higher as past studies have indicated that parents perceive schools as credible sources of nutrition information^(11)^, and Australian parents have indicated a desire for nutrition resources from schools^(37)^. On the other hand, previous national and international studies also demonstrated the dissatisfaction of parents with school-provided resources. For example, in Gaspar’s study (I, Gaspar, dissertation, 2020) with Swiss parents, participants reported receiving a few nutrition information brochures from school, finding them insufficient for implementing dietary changes similar to some Australian parents in a study who regarded school resources as not useful^(37)^. Considering study findings and previous research, it can be concluded that well-structured impactful food and nutrition resources created by nutrition professionals and promoted through schools have the potential to serve as trustworthy and easily accessible tools for parents^(38)^. The creation of such resources by nutrition professionals would not only reduce the burden on schools but also address food and nutrition expertise needs at schools.

Non-native English-speaking and younger parents were more interested in receiving food and nutrition information to improve their children’s eating behaviours. Non-native English-speaking parents may encounter difficulties accessing health information due to language barriers, cultural differences, and unfamiliar ways of navigating information sources and healthcare systems^(39)^. Additionally, the process of acculturation, which involves the exchange of immigrants’ original attitudes and behaviours with those of the host culture^(40)^, may also have a negative impact on their families’ healthy eating habits, including those of their children. As a result, these parents may face more significant challenges in providing healthy meals for their children. Therefore, the provision of culturally appropriate resources for parents is required may be worthwhile. It was also not unexpected that younger parents would be more interested in receiving food and nutrition-related information because nutritional knowledge has been shown to increase with age^(41)^. In light of these considerations, it might be wise to prioritise parent groups with non-Australian backgrounds as well as younger parent groups in the provision of food and nutrition information.

Universalism-concern value score was significantly and positively associated with parents’ wanting to receive food and nutrition-related information from their child’s school. The universalism-concern value is about equality and social justice^(23)^. Primary school is a suitable venue to ensure equity among children and parents due to its wide reach^(42)^. Therefore, it is not surprising that parents with higher universalism-concern value scores were more likely to prefer schools to provide food and nutrition-related information. These findings highlight that individuals’ personal values play a more significant role in shaping their views than many of their demographic characteristics. Designing interventions that only take into account target populations’ demographic characteristics may not provide favourable outcomes. The exploration of their values is equally important. It is worth noting that values, including universalism, can be more easily altered compared with stable demographic characteristics and can be impacted through various methods, such as communication campaigns^(43)^.

Parents mainly preferred online dissemination of food and nutrition information; the least preferred method was weekend workshops. However, these findings are in contrast with the findings from a previous qualitative study by the authors^(19)^, in which parents stated they would be willing to attend a workshop at school. This could be because parents who volunteer for hour-long interviews may be more interested in food and nutrition than the broader group of parents who responded to the short survey. In addition, the previous study was conducted prior to and during the first months of the COVID-19 lockdown. It might be argued that the COVID-19 pandemic changed parents’ preferences in this regard (the survey was conducted in the second year of the COVID pandemic lockdown with extended significant lockdowns in most parts of Australia). During the peak times of the pandemic, face-to-face events were replaced with online versions, and people became more accepting of this kind of delivery.

On the other hand, consistent with parents’ preference for online delivery instead of face-to-face meetings, low parental attendance at workshops because of scheduling and transportation difficulties has been widely reported^(44)^. In addition, Jones *et al.* ^(45)^ demonstrated Australian parents’ high acceptance of online nutrition education, where recruitment rates were higher and attrition rates lower comparatively. It is recommended to consider using online education delivery methods to enhance parent involvement^(46)^. The use of online interventions may address the issues experienced by working parents with busy lifestyles, which can be a barrier to joining health programmes^(47)^. It is important to note that parents’ preferred channels for food and nutrition education may vary significantly and may be associated with their demographic characteristics^(38,48)^. Past research has highlighted that parents predominantly obtain nutrition information from a variety of sources. These include traditional printed resources like books, recipes, research articles, health magazines and booklets. Additionally, parents engage with media such as television and radio, utilise Internet-based platforms like websites and online parenting forums or blogs, and seek professional guidance from sources like nurses, dietitians, and childcare staff. Furthermore, they rely on an informal support network, encompassing mother’s groups, family, friends and fellow parents^(38,48)^. Therefore, future resources should be made accessible to parents through various formats, including online and printed booklets, on a website or in a face-to-face workshop^(48)^. This strategy would enable wide reach and increase the effectiveness of interventions. In addition, the Australian Guide to Healthy Eating (AGHE) may be a useful and relevant resource to parents, suggesting a need for increased efforts and resources in promoting AGHE and integrating it into interventions and programmes designed for parents.

Parents in this study were interested to know more about ways to encourage healthy eating among their children. This finding concurs with the existing literature. Previous research on parental knowledge and the use of dietary guidelines indicate that parents usually have a good understanding of what they should feed their children. However, there is a significant gap in understanding how to motivate their children to eat healthily^(48)^. Current research suggests that parents need ideas and support on how to discuss healthy eating and encourage their children to eat healthily^(49)^. Current research suggests that parents require practical suggestions and guidance on how to discuss healthy eating and encourage their children to eat healthily. Labelling their children as fussy or picky was common, similar to existing international studies in which up to 50 % of mothers reported that their child was difficult to feed or picky^(50)^. Similar to Australian preschool parents^(48)^, parents in the current study wanted information to deal with fussy eating with minimal stress. This result may not be surprising as pickiness has been shown to be stable from preschool to school age^(51)^.

Moreover, parents in the current study desired to learn more about lunchbox ideas, food labels and age-specific portion sizes and recommendations. These findings may not be unique to Australia. For example, Gaspar (I, Gaspar, dissertation, 2020) demonstrated that parents from Switzerland struggled the most with the more complex aspects of dietary intake, including food labels and recommended daily servings, despite having a basic comprehension of what constitutes a nutritious diet. In addition, previous international and national studies have shown that parents want specific and applicable information, such as portion sizes for different age groups and information that pertains to their child’s nutritional needs^(47,52–54)^ and lunchbox ideas^(37)^. Therefore, future public health interventions and programmes should include these topics to address parents’ needs to facilitate their efforts to improve their children’s nutrition.

The study identified some demographic characteristics of the parents who demonstrated a lower inclination to seek knowledge about food and nutrition specifically noting a correlation with an English-speaking background and older age. However, other influencing factors, such as their existing knowledge and prior experiences with provided resources, might have contributed to their perspectives. For instance, Hart’s study^(48)^ on Australian parents who expressed disinterest in healthy eating resources revealed that they perceived their knowledge or informal support networks as already sufficient. Similarly, in our study, parents might have considered their existing knowledge or support networks satisfactory. In addition, in a study by Gaspar (I, Gaspar, dissertation, 2020) with Swiss parents, participants found nutrition information brochures from schools to be inadequate for implementing dietary changes. They also voiced reluctance to receive more printed materials, deeming them ineffective (I, Gaspar, dissertation, 2020), a sentiment echoed by Australian parents in another study^(37)^ who viewed school resources as unhelpful. To better inform public health strategies, further research is warranted to investigate the reasons behind parental disinterest in receiving food and nutrition information.

### Strengths and limitations

The main strength of this study was that it included various demographic groups across Australia explored parents’ needs for food and nutrition-related information. The findings are novel as this is the first survey to explore parents’ views of their own food and nutrition information needs. Consistent with the convergent design approach, both qualitative and quantitative data were gathered, and each dataset was analysed independently. The results of both analyses were then interpreted together, providing a more comprehensive understanding of the views of contemporary Australian parents. Some limitations of this study need to be considered when interpreting the findings. First, causal relationships between the variables could not be examined due to the cross-sectional nature of the study. Second, the study may have attracted individuals who have a greater interest in food and nutrition, which could have resulted in a selection bias. Third, the sample was skewed towards high and mid-SES and more university-educated compared with the Australian average. Therefore, it is crucial for future studies to actively seek out and include demographic groups that may have been underrepresented in the current investigation. Lastly, the extensive length of the survey hindered the incorporation of a tool to evaluate parents’ existing knowledge concerning food and nutrition which might have an impact on their preferences to learn more about food and nutrition. Addressing this gap in future investigations would contribute valuable insights into understanding and addressing potential disparities in parents’ food and nutrition information requirements.

### Implications for practice

These findings can inform public health educators and assist them in designing future food and nutrition education programmes and resources for primary school parents. Parents should have access to food and nutrition-related resources, including topics they identified in the study to develop their own knowledge and self-efficacy regarding food and nutrition. These can be designed by nutritionists or public health educators and facilitated by primary schools, as most parents regarded schools as a credible venue. Various communication channels, including online/printed newsletters and YouTube, should be used for the dissemination of food and nutrition resources or delivery of the relevant training. Lastly, as the main language spoken at home and age were strong factors of whether parents would like to learn more about food and nutrition, young parent groups and parents with non-Australian backgrounds should be prioritised in the provision of food and nutrition information.

## Supporting information

Aydin et al. supplementary material Aydin et al. supplementary material

## Data Availability

The data that support the findings of this study are available on request from the corresponding author. The data are not publicly available due to privacy or ethical restrictions.

## Supplementary material

For supplementary material accompanying this paper visit https://doi.org/10.1017/S1368980024000387
