# Supplementary material for: Food and nutrition information requirements of Australian primary school parents
Source: Public Health Nutr. 2024 Feb 5;27(1):e65. doi: 10.1017/S1368980024000387 (PMC10897573; doi:10.1017/S1368980024000387)
Supplement: Aydin et al. supplementary material [file S1368980024000387sup001.docx]

**Demographic Characteristics**

|  | **n (%)** |
| --- | --- |
| ***Gender***(N=787) |  |
| Female | 757 (96) |
| Male | 26 (3) |
| Prefer not to say | 4 (1) |
| Non-binary | 0 (0) |
| ***Marital Status*** (N=787) |  |
| Married/de facto | 678 (86) |
| Single/divorced/widowed | 91 (12) |
| Prefer not to say | 18 (12) |
| ***Education***(N=787) | |
| Postgraduate degree | 242 (31) |
| University degree | 323 (41) |
| Trade/certificate/apprentice | 152 (19) |
| Year 12 or less | 70 (9) |
| ***Age group (years)*** (N=785) |  |
| 20-29 | 36 (5) |
| 30-39 | 368 (47) |
| 40-49 | 346 (44) |
| 50+ | 35 (4) |
| ***Child Grade*** (N=787) |  |
| Prep | 169 (21) |
| Grade 1 | 149 (19) |
| Grade 2 | 113 (14) |
| Grade 3 | 107 (14) |
| Grade 4 | 76 (10) |
| Grade 5 | 86 (11) |
| Grade 6 | 87 (11) |
| ***State of residency***(N=786) |  |
| ACT | 23 (3) |
| NSW | 89 (11) |
| NT | 9 (1) |
| QLD | 54 (7) |
| SA | 16 (2) |
| TAS | 8 (1) |
| VIC | 437 (56) |
| WA | 150 (19) |
| ***Remoteness**** (N=786) |  |
| Major Cities | 517 (66) |
| Rural and remote areas | 269 (35) |
| ***SES level***** (N=786) |  |
| High-SES | 425 (54) |
| Mid-SES | 292 (37) |
| Low-SES | 69 (9) |
| ***Main language* spoken at home (**N=787) |  |
| English | 728 (93) |
| Other | 59 (8) |
